# The optimal cut-off value of postoperative day three C-reactive protein to predict for major complications in colorectal cancer patients

**DOI:** 10.1007/s00423-025-03655-2

**Published:** 2025-02-27

**Authors:** Coco Smit, Maryska L. Janssen-Heijnen, Frits van Osch, Jonas Rops, Anke H. C. Gielen, Maarten van Heinsbergen, Jarno Melenhorst, Joop L. M. Konsten

**Affiliations:** 1 Department of Surgery, VieCuri Medical Centre, Venlo, The Netherlands; 2 GROW School for Oncology and Developmental Biology, Maastricht, The Netherlands; 3 Department of Clinical Epidemiology, VieCuri Medical Centre, 5912 BL Venlo, The Netherlands; 4 Department of Epidemiology, GROW Research Institute for Oncology & Reproduction, Faculty of Health, Medicine and Life Sciences, Maastricht University, 6200 MD Maastricht, The Netherlands; 5 Maastricht University, Faculty of Health, Medicine and Life Sciences, Maastricht, The Netherlands; 6 Department of Surgery, Maastricht University Medical Centre, Maastricht, The Netherlands; 7 School of Nutrition and Translational Research in Metabolism (NUTRIM), Maastricht University, Maastricht, The Netherlands

**Keywords:** Colorectal cancer, Postoperative monitoring, Complications, CRP

## Abstract

**Purpose:**

To identify an optimal postoperative day 3 (POD3) C-reactive protein (CRP) cut-off for predicting major complications in colorectal cancer (CRC) patients. Secondary objectives included identifying patient and surgical factors associated with POD3 CRP levels and assessing the accuracy of the cut-off across subgroups.

**Methods:**

A retrospective cohort study of 1536 CRC patients who underwent an oncological resection was conducted. The predictive accuracy of POD3 CRP for major complications was tested using Receiver Operating Characteristics curves. The CRP cut-off was tested across subgroups. Multivariable logistic regression analyses was performed to evaluate the predictive value of the POD3 CRP cut-off, while also determining whether patient and surgical characteristics independently predicted major complications.

**Results:**

An optimal cut-off of 114 mg/L was identified, with a sensitivity of 0.80 and specificity of 0.59 and an Area Under the Curve for POD3 CRP of 0.78. Sensitivity remained consistently high across all subgroups, whereas specificity exhibited variability, with a notable decrease observed in the subgroups; aged 66–69, obese, ASA III and open surgery. After adjusting for patient and surgery characteristics, a POD3 CRP level above 114 mg/L was associated with a significant 5.29-fold increase in the odds for developing major complications.

**Conclusions:**

A POD3 CRP cut-off of 114 mg/L is an effective predictor of major complications following CRC surgery, supporting safe early discharge. The cut-off remains a reliable predictor, even after adjusting for patient and surgery factors.

**Supplementary Information:**

The online version contains supplementary material available at 10.1007/s00423-025-03655-2.

## Introduction

Colorectal cancer (CRC) remains a significant health burden, with approximately 17,000–18,000 new cases diagnosed annually in the Netherlands [1]. Surgical resection remains the cornerstone in curative treatment, with approximately 70% of affected patients undergoing surgical treatment [2].

Postoperative complications are reported in 20–40% of colorectal surgical procedures. These complications are associated with increased (hospital) morbidity and mortality [3–6]. Furthermore, these complications could prolong length of hospital stay and increase healthcare costs. Notably, up to 31% of hospital costs for CRC patients are linked to managing postoperative complications [7–9].

In response to these risks, various strategies have been implemented to enhance recovery and reduce complications. Laparoscopic surgery, combined with the Enhanced Recovery After Surgery (ERAS) protocol, has become a widely adopted approach in colorectal surgery [10]. The clinical goal is to achieve safe discharge between the third and fifth day after surgery [11–13]. However, we see a trend towards earlier discharge to avoid hospital acquired infections, improve Quality of Life (QoL) and reduce hospital costs.

One crucial marker in predicting postoperative complications is C-reactive protein (CRP). CRP has become an important tool in determining a patient’s readiness for safe and early discharge as it serves as a useful marker for detecting postoperative complications. However, several other factors can contribute to the elevation of CRP levels, for instance infection, inflammation, malignancy and trauma [14, 15]. In conjunction with the patient's clinical condition, body temperature, and other laboratory parameters such as white blood cell count (WBC), the postoperative day 3 (POD 3) CRP level determines the readiness of the patient for discharge and can assist to detect complications in patients who have had day care colorectal surgery. CRP is measured on POD 3 at these institutions because it typically peaks around this time due to a complication related inflammatory response. Although CRP levels are commonly elevated immediately following surgery, persistent or rising levels over time may indicate an underlying complication.

There is ongoing debate regarding the ability of CRP to predict the presence of major complications after colorectal surgery. Previous research has suggested a broad range of 152–190 mg/L as a potential threshold. [16–20]. Nowadays, hospital costs have to be reduced due to limited human and financial resources [21]. As we enter a new era aimed at faster patient discharge, high sensitivity and negative predictive value (NPV) are critical for preventing a premature discharge of patients with undetected complications. At the same time, maintaining reasonable specificity and positive predictive value (PPV) is essential to minimize unnecessary diagnostic procedures, which may lead to increased costs and patient burden.

Research concerning CRP and its role as a predictor of complications often includes various types of surgery, such as upper and lower gastro-intestinal surgery, limiting the ability to apply findings specifically to CRC patients. Additionally, despite the well-known susceptibility of CRP being affected by various factors, the recommended reference range for CRP has never been adjusted for variables such as patient and surgery characteristics.

This study aims to establish a cut-off value for CRP on POD 3, in order to predict major complications following colorectal surgery, while also evaluating whether this cut-off performs consistently across subgroups defined by patient and surgery characteristics. By refining predictive markers like CRP, we may be able to enhance postoperative monitoring and improve patient outcomes through timely intervention and safe early discharge in the future.

## Methods

### Study design and participants

A multicenter retrospective cohort study was conducted, including all patients who underwent an oncological colorectal resection in the peripheral teaching hospital VieCuri Medical Centre and the tertiary referral center for CRC, the Maastricht University Medical Center (MUMC +) in the Netherlands between January 2016 and December 2023.

Male and female adult patients (> 18 years of age) with CRC who underwent surgical resections at VieCuri or MUMC + were eligible for inclusion. Exclusion criteria were an age younger than 18, benign indications for colorectal resection and surgical procedures without (segmental) bowel resection such as colostomy construction only. Additionally, patients with missing POD 3 CRP data were excluded.

### Clinical parameters and data collection

Retrospective data from the electronic patient records were extracted and transferred to Castor. Recorded baseline characteristics included age at the time of surgery (classified as age ≤ 65, age 66–69, age 70–74, age ≥ 75), gender, weight (classified as normal weight (BMI < 25), overweight (BMI < 30), obese (BMI ≥ 30)), American Society of Anesthesiologist (ASA) classification, smoking and alcohol status (classified as non-smoker, former smoker, active smoker; non-alcohol consumer, consuming less than one unit per day, consuming more than one unit per day).

Recorded surgical characteristics included type of surgery (open, laparoscopic, conversion, or robot-assisted) and specific procedure (group 1; hemicolectomy right or transverse colon resection, group 2; hemicolectomy left or sigmoid, group 3; rectal resections). This categorization is based upon the observation that transverse colon resection surgeries are less frequent and usually treated as extended right hemicolectomies. Comparatively, left hemicolectomy and sigmoid surgeries exhibit analogous intervention characteristics, while rectal surgeries tend to manifest greater complications, notably an increased incidence of anastomotic leakage [22].

Extracted laboratory findings included POD 3 CRP and leukocyte count. Recorded outcome included the postoperative complication score (identified on or after POD 3, within 30 days after surgery), classified according to Clavien-Dindo (Table 5, Appendix) [23]. Minor complications were defined as Clavien-Dindo Grade I and II, major complications as Clavien-Dindo ≥ Grade III.

### Statistical analysis

All statistical analyses were conducted using IBM SPSS Statistics (version 28; IBM Corporation, Armonk, NY, USA) and Python 3.12.2. A Shapiro–Wilk test revealed that the data were not normally distributed.

Baseline characteristics were presented as medians with interquartile ranges for continuous variables and as numbers with percentages for nominal variables. Statistical comparisons of baseline characteristics between the no/minor complication group and the major complication group were performed using either a Mann–Whitney U test for continuous data or a Pearson’s Chi-Squared test for nominal data.

To assess the accuracy of POD 3 CRP as a predictor for major complications, receiver operating characteristic (ROC) curves were generated for the total population as well as for subgroups based on patient and surgery characteristics, using scikit-learn version 1.5.0 and matplotlib version 3.9.0 in Python. Initial attempts to set a highly sensitive cut-off for POD 3 CRP resulted in unrealistic values with low specificity, which would lead to excessive testing, possibly unnecessary radiation, increased costs, and invasive procedures. In order to address this, a sensitivity of 0.80 was established as a threshold, aiming to ensure early yet safe discharge while accurately identifying patients at risk of major complications. Applying this sensitivity, the optimal POD 3 CRP cut-off value was defined from the ROC curve of the total population.

After determining the optimal cut-off value for the total population, it was tested across all subgroups based on patient and surgery characteristics by calculating the corresponding sensitivity, specificity, positive predictive value (PPV), and NPV.

Finally, univariable and multivariable logistic regression analyses were conducted to examine the predictive value of the determined POD 3 CRP optimal cut-off value for developing major complications. The multivariable logistic regression analysis also assessed whether patient and surgery characteristics were independent predictors of developing a major complication. A *p*-value of < 0.05 was considered statistically significant.

### Ethical approval

The study received a confirmation that it is not subject to the Medical Research Involving Human Subjects Act (WMO) from the Medical Ethics Committee of the University Hospital Maastricht (number 2022–3598). Local approval was obtained from VieCuri Medical Centre.

## Results

### Patient, surgery and laboratory characteristics

**Fig. 1:**
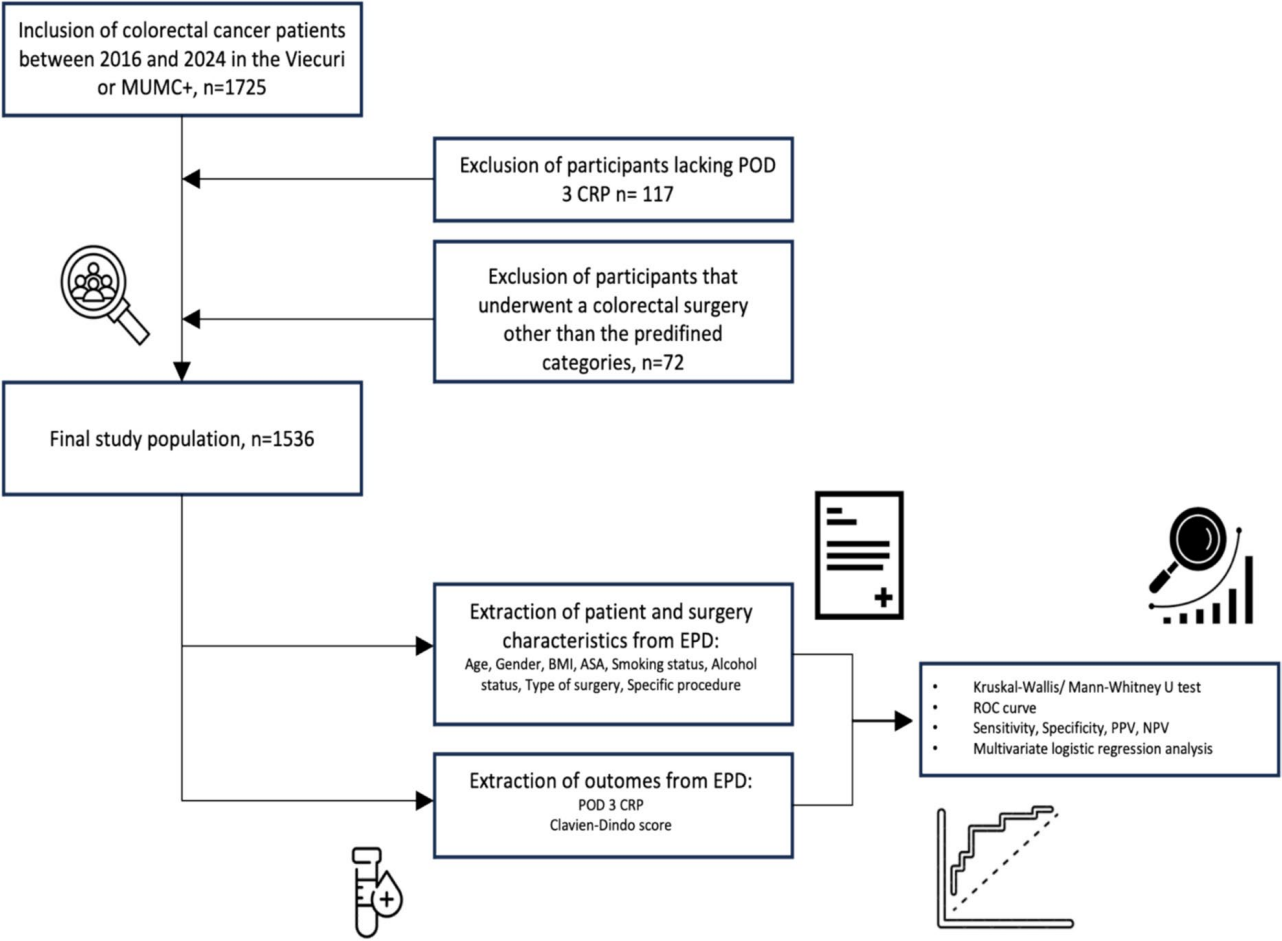
Flowchart presenting in- and exclusion criteria, patient and surgery characteristics and outcomes extracted from the electronic patient records and executed statistical analysis

Figure 1 illustrates that, following the exclusion of 117 patients without POD 3 CRP and 72 patients who underwent colon cancer surgery that did not fit any of our predefined categories (including procedures combining colorectal surgery with interventions in other anatomical regions, such as the ileum and hepatobiliary structures), a total of 1,536 patients were included in the study. Among these, 796 patients were included from VieCuri MC and 740 were from MUMC + .

Table 1 shows that the population consisted of 837 (54.5%) male and 699 (45.5%) female patients. In total, 615 (40.1%) patients underwent either a right sided hemicolectomy or transverse colon resection, 456 (29.7%) patients a left sided hemicolectomy or sigmoid resection and 463 (30.2%) patients a rectum resection. Laparoscopic resections were performed in 1004 (65.4%) patients, whilst 269 (17.5%) patients underwent primary open resection. In 195 (12.7%) patients, a primary laparoscopic resection had to be converted to an open procedure and 67 (4.4%) patients underwent a robot-assisted procedure.

**Table 1:** Patient/ surgery characteristics and laboratory findings for the total population, no/ minor complication group and major complication group

Variables	Total *N* = 1536	No/ Minor complication *N* = 1301	Major complication *N* = 235	*P*-value
Hospital^1^				0.3367^5^
VieCuri MC	796 (51.8)	681 (52.3)	115 (48.9)	
MUMC +	740 (48.2)	620 (47.7)	120 (51.1)	
Age^1^				0.617^7^
≤ 65	462 (30.1)			
66–69	231 (15.0)			
70–74	286 (18.6)			
≥ 75	557 (36.3)			
Gender^1^				0.157^5^
Male	837 (54.5)	699 (53.7)	138 (58.7)	
Female	699 (45.5)	602 (46.3)	97 (41.3)	
Smoking status^1^				**0.008**^7^
Non smoker	732 (49.9)	630 (50.7)	102 (45.5)	
Former smoker	557 (38.0)	476 (38.3)	81 (36.2)	
Current smoker	177 (12.1)	136 (11.0)	41 (18.3)	
Alcohol use^1^				0.064^7^
Non alcohol consumption	586 (39.9)	488 (39.3)	98 (43.8)	
< 1 glass a day	567 (38.7)	496 (39.9)	71 (31.7)	
≥ 1 glass a day	314 (21.4)	259 (20.8)	55 (24.6)	
BMI^1^				0.270^7^
Normal/ underweight	440 (33.9)	367 (38.5)	73 (36.1)	
Overweight	528 (40.7)	456 (41.6)	72 (35.6)	
Obese	329 (25.4)	272 (24.8)	57 (28.2)	
ASA classification^1^				** < 0.001**^7^
Grade I	115 (7.5)	108 (8.3)	7 (3.0)	
Grade II	912 (59.4)	797 (61.3)	115 (48.9)	
Grade III	492 (32.)	388 (29.8)	104 (44.3)	
Grade IV	17 (1.1)	8 (0.6)	9 (3.8)	
Surgical procedure^1^				**0.008**^7^
Hemicolectomy right and transverse colon resection	615 (40.1)	523 (40.3)	92 (39.1)	
Hemicolectomy left and sigmoid resection	456 (29.7)	402 (30.9)	54 (23)	
Rectum resection	463 (30.2)	374 (28.8)	89 (37.9)	
Type of procedure^1^				** < 0.001**^7^
Open	269 (17.5)	203 (15.6)	66(28.1)	
Laparoscopic	1004 (65.4)	891 (66.5)	113 (48.1)	
Conversion	195 (12.7)	152 (11.7)	43 (18.3)	
Robot	67 (4.4)	54 (4.2)	13 (5.5)	
Laboratory findings^2^				
POD 3 CRP^3^	109 (64–175)	99 (58.5–151)	214 (129–313)	** < 0.001**^6^
Leukocytes POD 3^4^	8.3 (6.6–10.4)	8.25(6.6–10.2)	8.9 (6.8–12)	**0.001**^6^

^1^Data presented as number (%), ^2^Data presented as median (IQR), ^3^(mg/ L), ^4^(10^9^/L), ^5^Fisher’s Exact test, ^6^Mann-Whitney U-test, ^7^Pearson’s Chi-Squared test

**Fig. 2:**
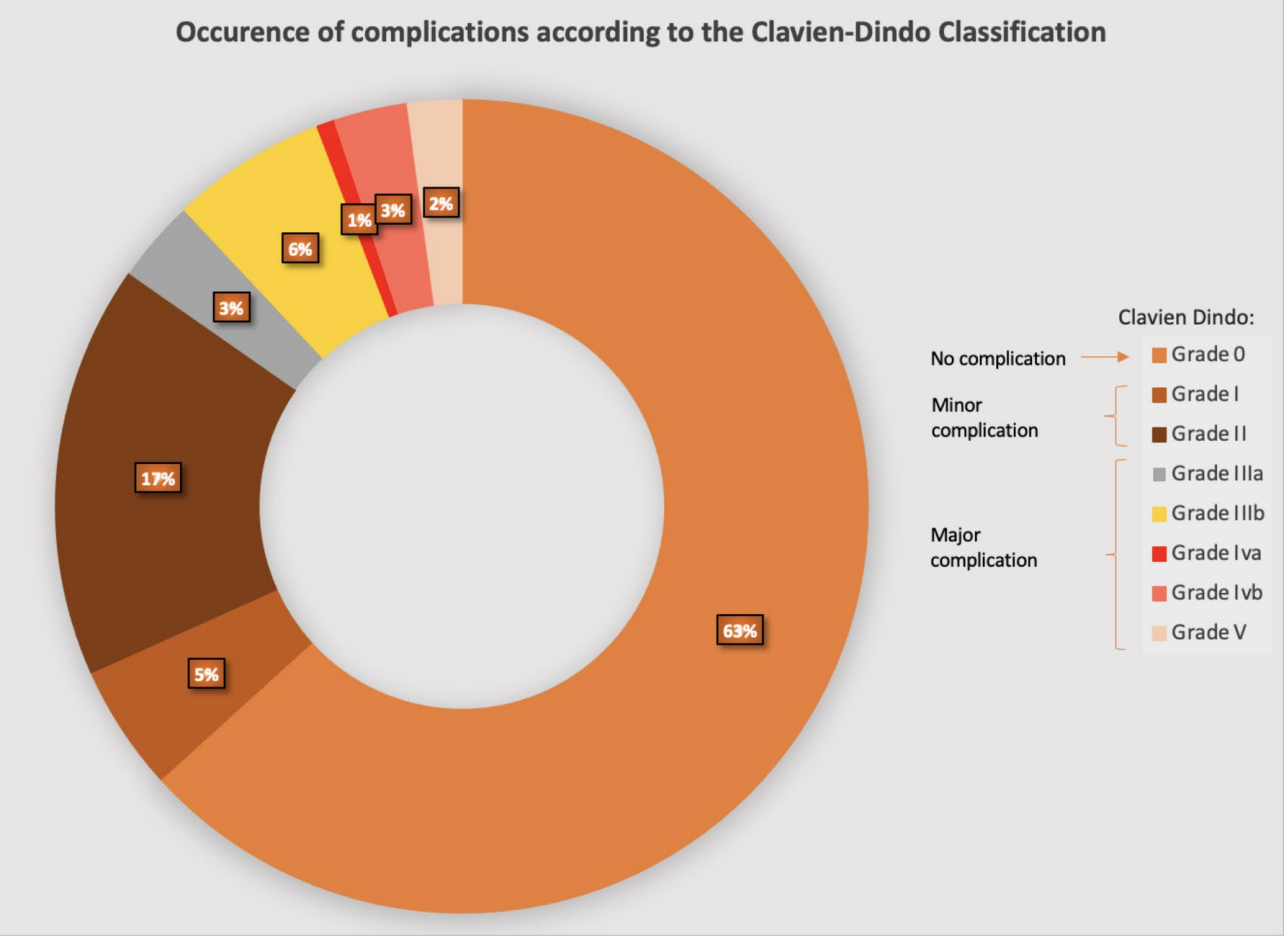
Graphical representation of complications according to the Clavien-Dindo classification

A total of 972 (63.3%) patients recovered without any post-operative complications, 328 (21.4%) patients developed a minor complication, whereas 235 patients (15.3%) experienced a major complication (Fig. 2).

A median POD 3 CRP for the group with no or minor complication of 99 mg/L (58.5–151) was found, whereas a median POD 3 CRP of 214 mg/L (129.0–313.0) was found for the group with a major complication. A median POD 3 white blood cell count for the group with no or minor complication of 8.25*10^9^/L (6.6–10.2) was found, whereas a median of 8.9*10^9^/L (6.8–12.0) was identified for the group with a major complication.

No statistically significant differences in age, gender, alcohol consumption and body mass index (BMI) distribution were found between the no or minor complication group and the major complication group. Conversely, statistically significant differences were observed in the distribution of smoking status, ASA classification, type of surgery, and specific procedures between the no/minor complication group and the major complication group. The major complication group had a higher percentage of smokers and a lower proportion of ASA I/II patients, with a greater representation of ASA III/IV patients. Additionally, this group showed a higher percentage of rectal surgeries, more open or converted procedures, and fewer laparoscopic operations.

### ROC curve for the total population and optimal cut-off value

**Fig. 3:**
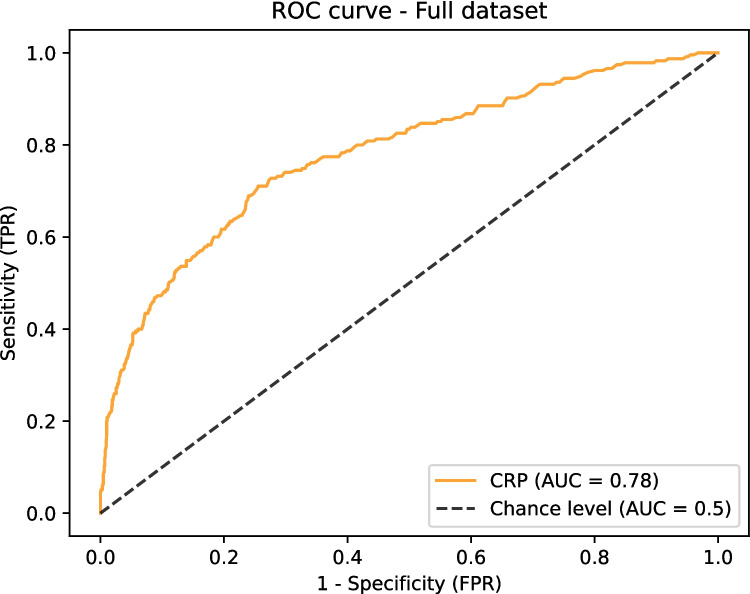
ROC curve with corresponding AUC for the total population

The diagnostic accuracy of POD 3 CRP with regard to development of a major complication is displayed in Fig. 3 by ROC curve. The ROC curve of POD 3 CRP to predict major complications for the total population shows a mean AUC of 0.78. An optimal POD 3 CRP cut-off value of 114 mg/L was identified for the total population to achieve a sensitivity of 0.80 (95% CI: 0.74–0.85), with a corresponding specificity of 0.58 (95% CI: 0.56–0.61), PPV of 0.26 (95% CI: 0.24–0.27), and a NPV of 0.94 (95% CI: 0.93–0.95). A higher sensitivity was not achievable with a clinically realistic cut-off point and would compromise specificity.

### Sensitivity, specificity, PPV and NPV of POD 3 CRP optimal cut-off value 114 mg/L for predicting major complications

**Fig. 4:**
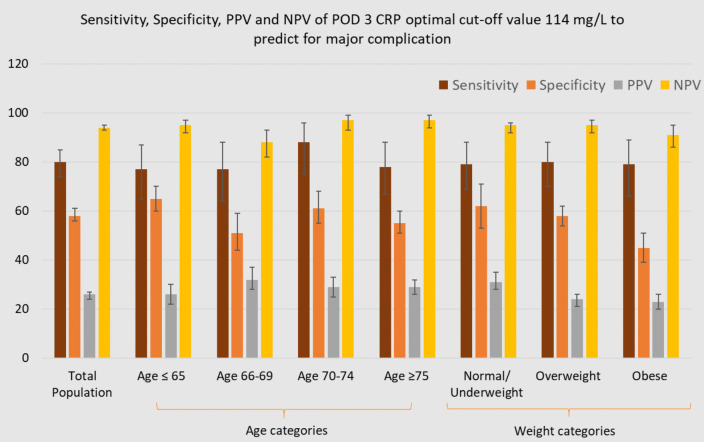
Diagnostic accuracy of POD 3 CRP cut-off 114 mg/L for predicting major complications within subgroups

**Fig. 5:**
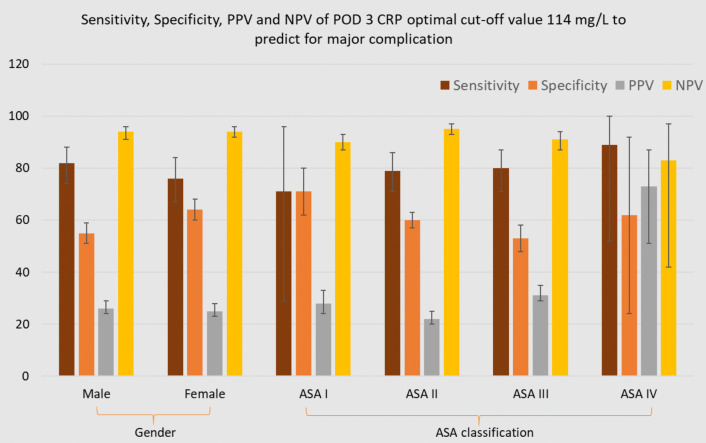
Diagnostic accuracy of POD 3 CRP cut-off 114 mg/L for predicting major complications within subgroups

**Fig. 6:**
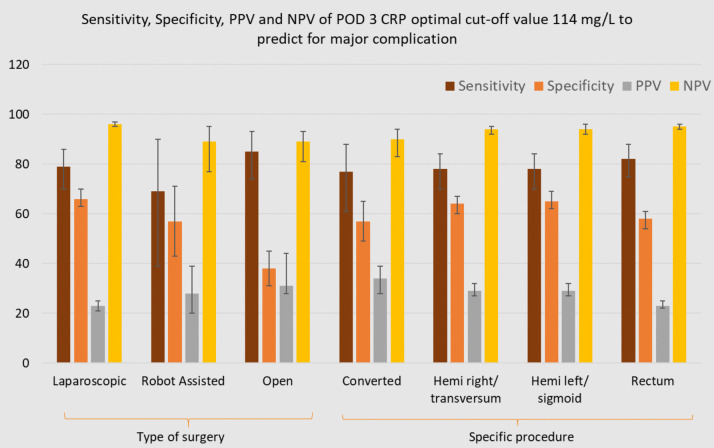
Diagnostic accuracy of POD 3 CRP cut-off 114 mg/L for predicting major complications within subgroups

Figures 4, 5 and 6 illustrate the sensitivity, specificity, PPV and NPV of the optimal POD 3 CRP cut-off value (114 mg/L) for predicting major complications, stratified by patient and surgical characteristics.

When applying the optimal POD 3 CRP cut-off value of 114 mg/L across specific subgroups, sensitivity remained consistently high, ranging from 0.71 (95% CI: 0.29–0.96) in ASA I to 0.89 (95% CI: 0.52–1.00) in ASA IV. In contrast, specificity showed a notable decrease in subgroups such as Age 66–69 (0.51, 95% CI: 0.44–0.59), obese patients (0.45, 95% CI: 0.39–0.51), ASA III patients (0.53, 95% CI: 0.48–0.59), and those undergoing open procedures (0.38, 95% CI: 0.31–0.45), compared to the specificity observed in the entire population (95% CI: 0.56–0.61). NPV ranged from 0.83 (ASA IV) to 0.97 (laparoscopic/age 66–69). The PPV ranged from 0.22 (95% CI: 0.19–0.25) in ASA II and age ≥ 75 subgroups to 0.73 (95% CI: 0.51–0.87) in ASA IV.

All ROC curves for patient and surgery characteristic-specific subgroups are presented in the Appendix, Fig. 7a-t. Additionally, the optimal POD 3 CRP cut-off values for each subgroup, determined for a sensitivity of 0.8, along with the corresponding specificity, PPV and NPV are presented in the Appendix, Table 3. The optimal cut-off value for one specific subgroup was particularly noteworthy, namely the open surgery group, where a cut-off value of 150 mg/L was identified. Additionally, relatively low optimal cut-off values were found for the ASA I, robot-assisted, and rectum groups, with values of 66, 84, and 91 mg/L, respectively.

### Univariable and multivariable logistic regression analysis

Univariable logistic regression analysis demonstrated that exceeding the optimal cut-off value (114 mg/L) results in 5.58 times (95% CI: 3.99–7.80, p < 0.001) the odds of developing a major complication compared to a POD 3 CRP below 114 mg/L (Table 2).

**Table 2:** Univariable and Multivariable Logistic Regression Analysis for predicting major complications with POD 3 CRP cut-off of 114 mg/L

Variables	Univariable OR (95% CI)	*P*- value	Multivariable OR (95% CI)^1^	*P*-value
Cutoff Value POD 3 CRP > 114 mg/L	5.58(3.99–7.80)	** < 0.001**	5.29(3.55–7.90)	** < 0.001**

^**1**^Adjusted for age categories, gender, weight categories, smoking and alcohol status, ASA classification, type of surgery, and specific procedure

Multivariable logistic regression analysis identified significant predictor variables among all patient and surgery characteristics. Age 66–69, obesity, ASA III, ASA IV, open, conversion and rectal surgery were identified as significant independent predictors of major complications, with obesity being the only significant negative predictor (Table 4, Appendix). Moreover, multivariable logistic regression indicated that exceeding a POD 3 CRP level of 114 mg/L was associated with a 5.29-fold increase in the risk of developing a major complication (95% CI: 3.55–7.90, *p* < 0.001) (Table 2).

## Discussion

The primary objective of this multicenter retrospective cohort study was to determine the optimal POD 3 CRP cut-off value for predicting major complications after CRC surgery. Particular emphasis was placed on identifying patients with major complications before discharge, by selecting a high-sensitivity threshold, in anticipation of shorter hospital stays in the future.

An optimal POD 3 CRP cut-off value of 114 mg/L was identified to predict major complications after CRC surgery, with a sensitivity of 0.80, specificity of 0.59, PPV of 0.26, and NPV of 0.94. This indicates that 94% of patients with a CRP level below 114 mg/L are correctly identified as not having major complications, supporting early discharge without compromising patient safety.

When applying this cut-off to specific subgroups, sensitivity remained high across all categories, although specificity varied, being notably lower in groups such as patients aged 66–69, obese patients, ASA III patients and those undergoing open procedures. The lower specificity observed in certain subgroups can be attributed to several factors. Obese patients, for instance, may have higher preoperative CRP levels irrespective of complications, thereby reducing the distinction between those with and without complications [24]. Similarly, patients classified as ASA III, who typically present with greater comorbidity burden, often exhibit elevated preoperative CRP levels [25, 26]. Furthermore, individuals undergoing open surgical procedures experience heightened postoperative inflammation and thus present a higher POD 3 CRP due to more extensive tissue trauma compared to minimally invasive surgery [27].This could also be a potential explanation for the finding of a relatively high optimal cut-off value of 150 mg/L observed in the open surgery subgroup. The broad range of PPVs can be explained by the relatively small sample sizes within each population subgroup, thereby limiting the precision and generalizability of the findings.

Age, obesity, ASA classification, type of surgery and specific procedure were identified as significant predictors for major complications after CRC surgery. After adjusting for various patient and surgical characteristics, an elevated POD 3 CRP level exceeding 114 mg/L remained significantly associated with 5.29-fold increased odds of major complications. This suggests that despite the potential influence of diverse patient and surgical characteristics on the predictive ability of POD 3 CRP for major complications, it continues to be a reliable diagnostic marker. This finding is consistent with the results of Straatman et al., who also concluded that, irrespective of patient characteristics (such as gender, age, and BMI) and the surgical techniques employed, postoperative CRP levels can be used to predict major postoperative complications [28, 29].

To minimize the influence of various patient and surgical characteristics and to more precisely assess the occurrence of complications, one could consider using the trend of CRP as a diagnostic marker (e.g., the difference between CRP levels on postoperative day 2 and day 3). Previous studies have demonstrated that an increase of ≥ 50 mg/L between any two postoperative days, regardless of the absolute CRP value, is predictive of the occurrence of complications [30, 31].

With the current trend toward earlier discharge, including trials exploring discharge within 23 h after CRC surgery, early identification of patients at increased risk for major complications is crucial. Our identified cut-off value will aid in this process; however, a key challenge remains in determining how to integrate this into clinical practice. At present, patients in our hospitals are admitted until at least POD 3. Keeping patients solely for this assessment is not a justifiable approach. In the future, monitoring could involve outpatient consultations on POD 3, or even the use of advanced wireless patches to track vital signs or potentially even CRP levels [32, 33].

Moreover, increasing research focuses on the diagnostic value of CRP on postoperative day 2 (POD 2), with a cut-off value of 170 mg/L, as proposed by A. Gielen et al. [17], although this approach applies a lower sensitivity. An alternative strategy could involve measuring POD 2 CRP in an outpatient consultation setting and, in cases of uncertainty, repeating the measurement on POD 3. This approach would allow for the assessment of the previously mentioned clinically relevant difference in CRP levels while minimizing the influence of patient and surgical characteristics.

This study represents the largest investigation to date on the relationship between patient and surgical characteristics and POD 3 CRP levels for predicting major complications. Additionally, it is the first to assess the utility of this cut-off value across various subgroups, filling an important gap in the current literature. The inclusion of patients from both peripheral and academic hospitals in equal proportions further strengthens its external validity. Additionally, the study's exclusive focus on CRC patients, excluding those with benign conditions or requiring HPB or upper GI surgery, may differentiate it from previous research.

However, its retrospective design limits the ability to establish causality. While the overall sample size is substantial, smaller subgroup sizes reduce result precision. Future research with larger subgroups is needed to refine the optimal cut-off value and improve reliability.

To develop an even more accurate diagnostic tool, future efforts may consider constructing a prediction model that integrates variables such as POD 3 CRP levels, POD 3 white blood cell count, body temperature (fever), heartrate (tachycardia) and patient and surgical characteristics with special emphasis on specific procedure (minimally invasive y/n) and ASA classification (high ASA (III/IV) y/n). Such a model could calculate the personalized odds of developing a major complication after colorectal cancer surgery.

## Conclusion

This retrospective cohort study identified an optimal POD 3 CRP cut-off value of 114 mg/L for predicting major complications, with an emphasis on facilitating early and safe postoperative discharge for CRC patients. The established cutoff value serves as a diagnostic marker for major complications in daily practice, notwithstanding the potential influence of varying patient and surgical characteristics.

Future research should focus on refining cut-off accuracy with larger subgroup samples and work towards comprehensive prediction models to enhance the accuracy of predicting a major complication in postoperative care for CRC patients and thereby work towards early, but especially safe discharge. Furthermore, clinicians could prioritize the change in CRP levels between consecutive measurements rather than relying on a single value to minimize the previously mentioned influence of patient and surgical characteristics.

## Supplementary Information

Below is the link to the electronic supplementary material.

Supplementary file1 (DOCX 1532 KB)

## Data Availability

No datasets were generated or analysed during the current study.
