# Supplementary material for: The optimal cut-off value of postoperative day three C-reactive protein to predict for major complications in colorectal cancer patients
Source: Langenbecks Arch Surg. 2025 Feb 27;410(1):85. doi: 10.1007/s00423-025-03655-2 (PMC11868158; doi:10.1007/s00423-025-03655-2)
Supplement: Supplementary file 1 — Supplementary file1 (DOCX 1532 KB) [file 423_2025_3655_MOESM1_ESM.docx]

## **Appendix**


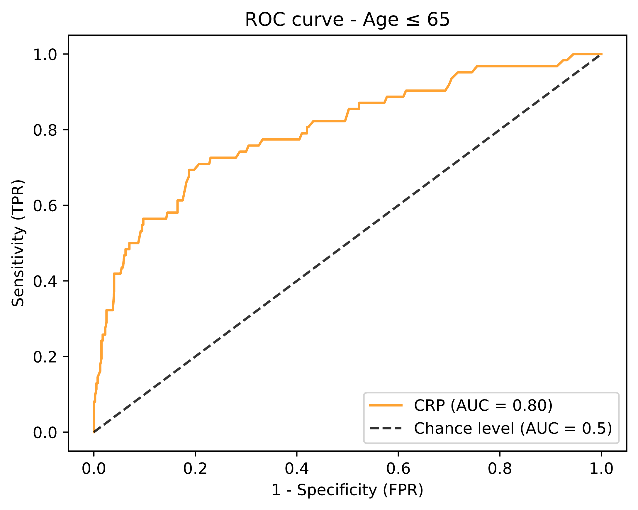

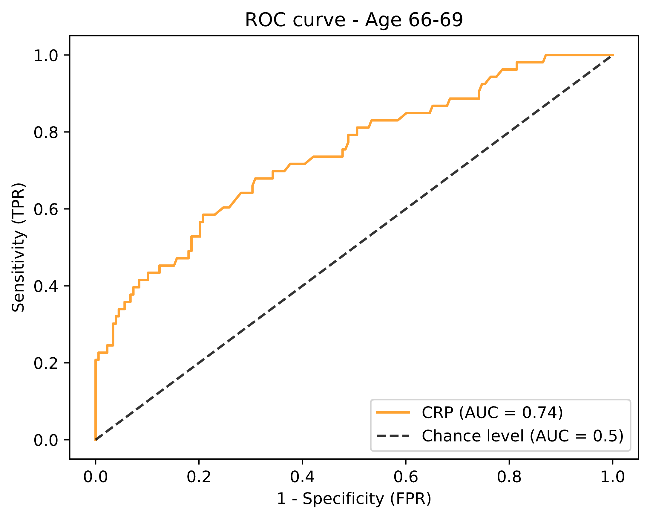


a. b.


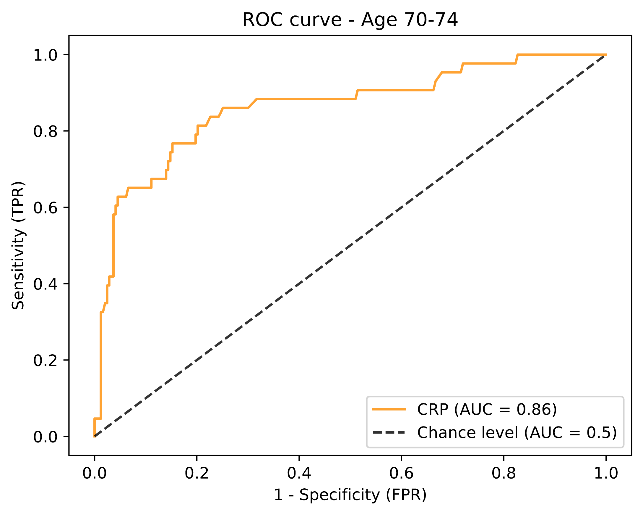

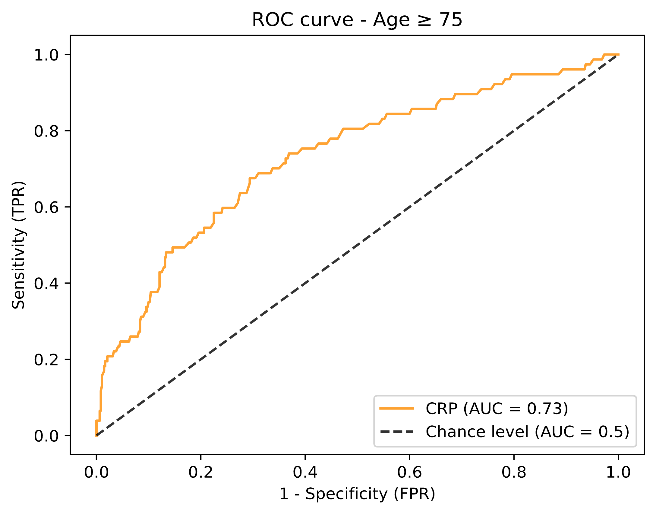


c. d.


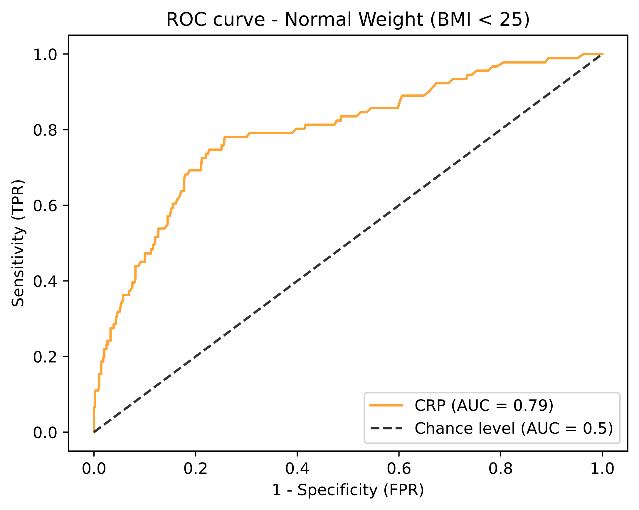

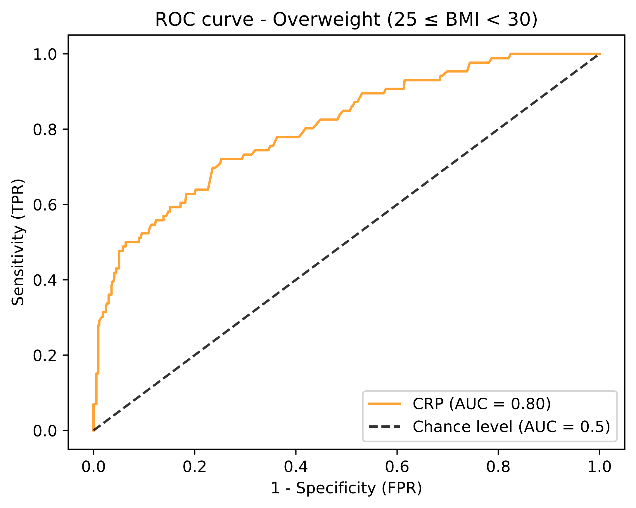


e. f.


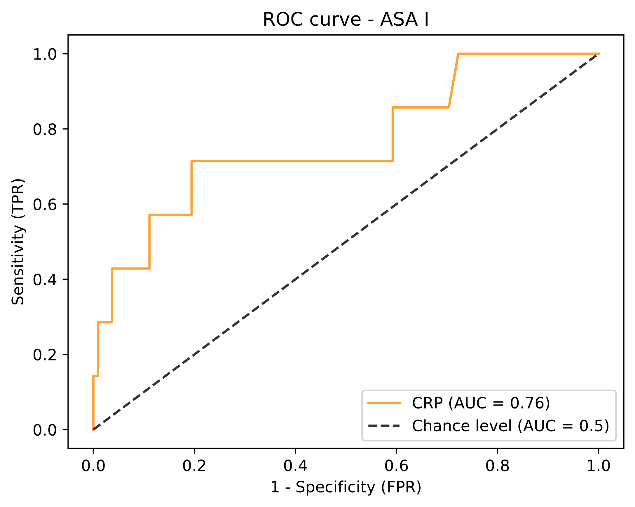

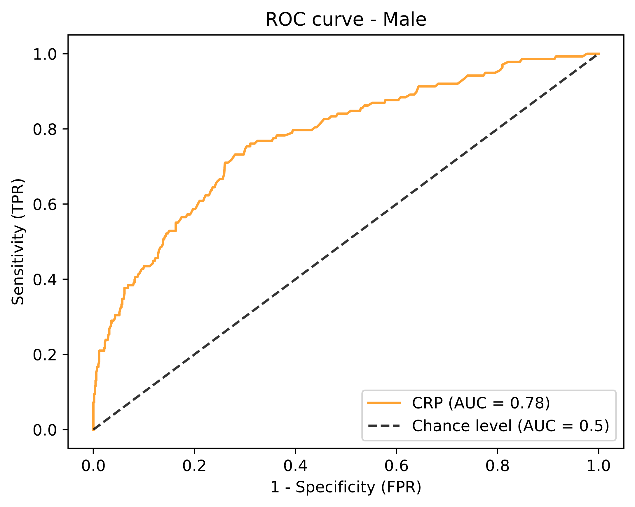

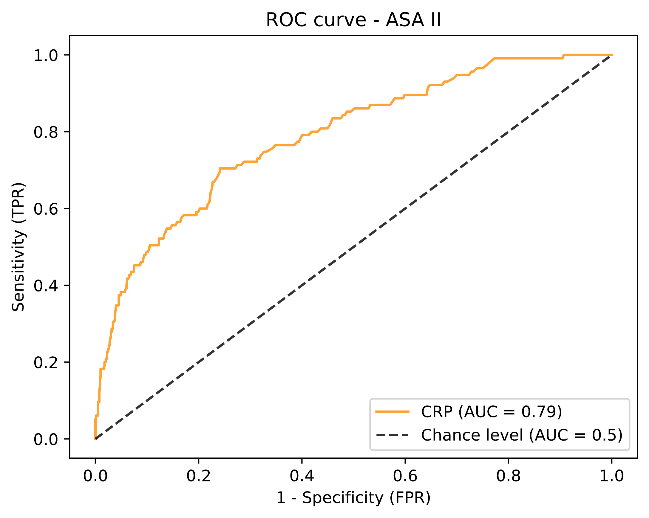

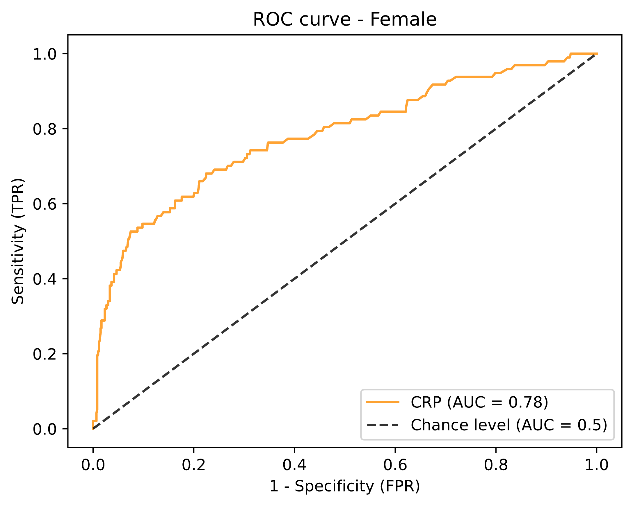
g. h.
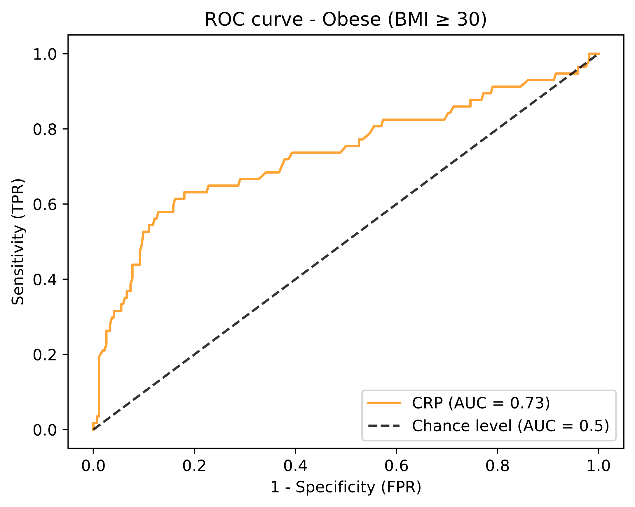


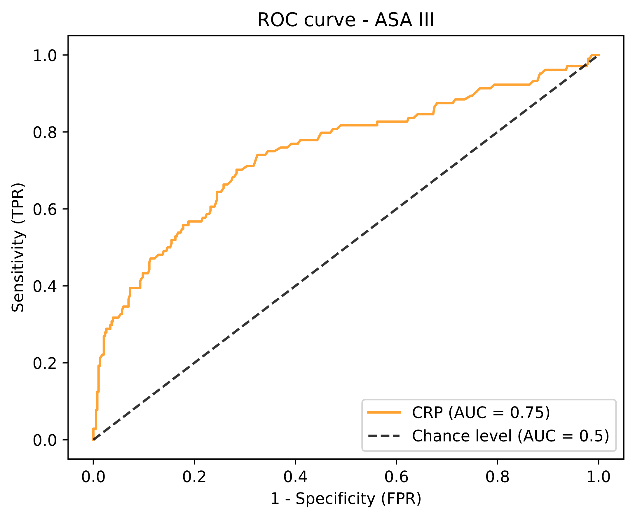
i. j.

k. l.


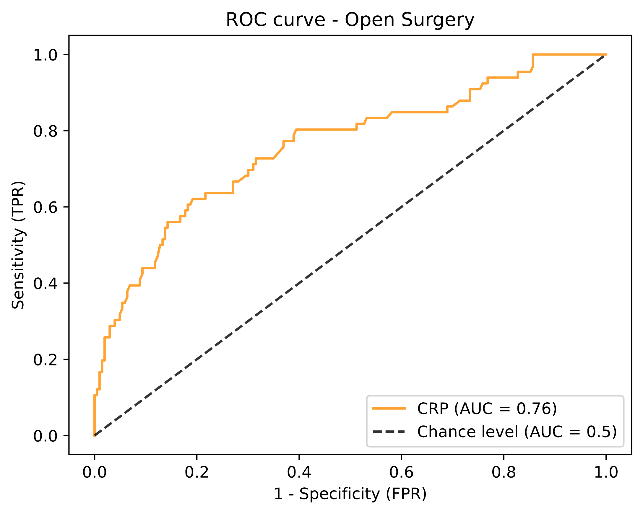

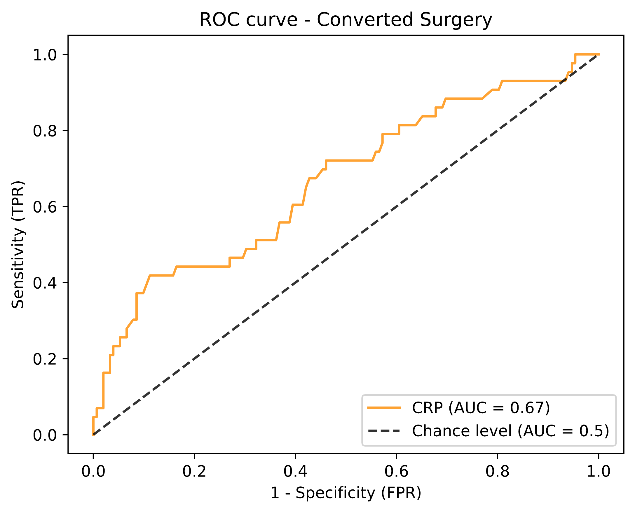

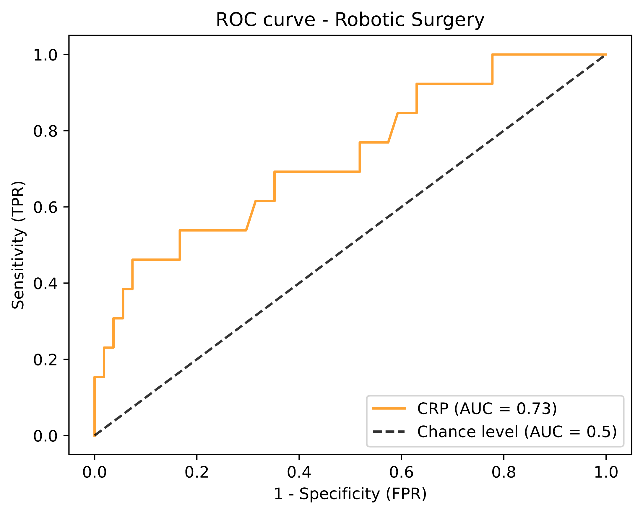

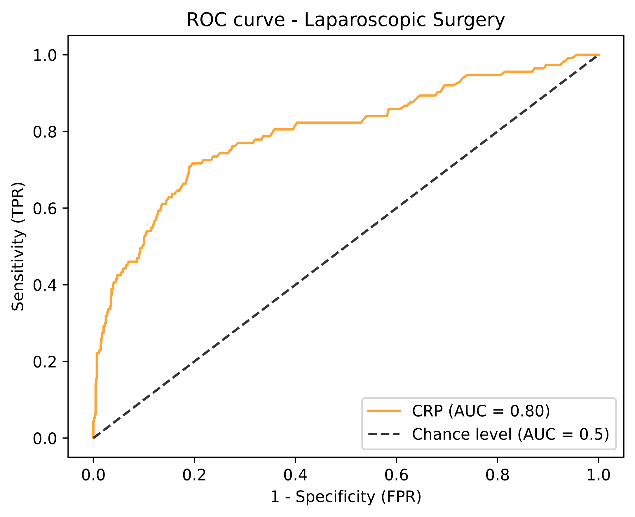

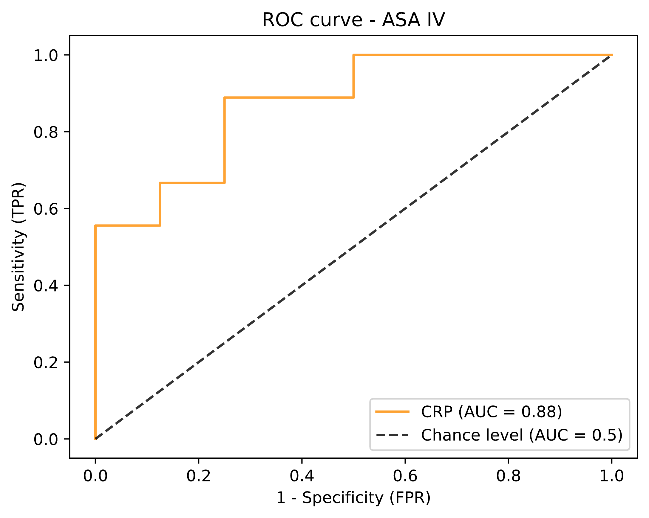
m. n.


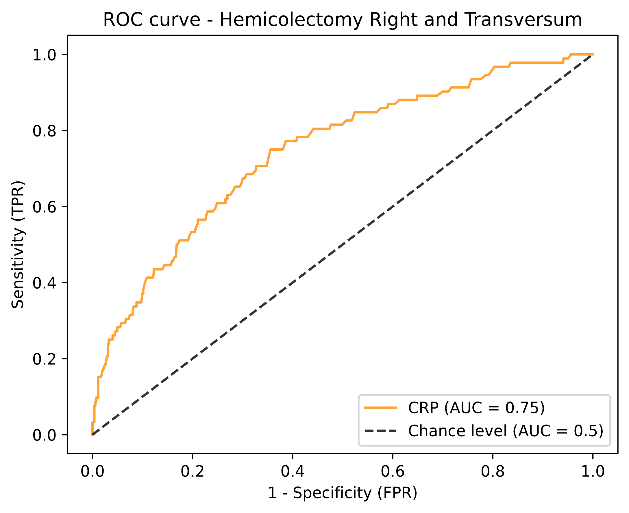
o. p.

q. r.

s. t.
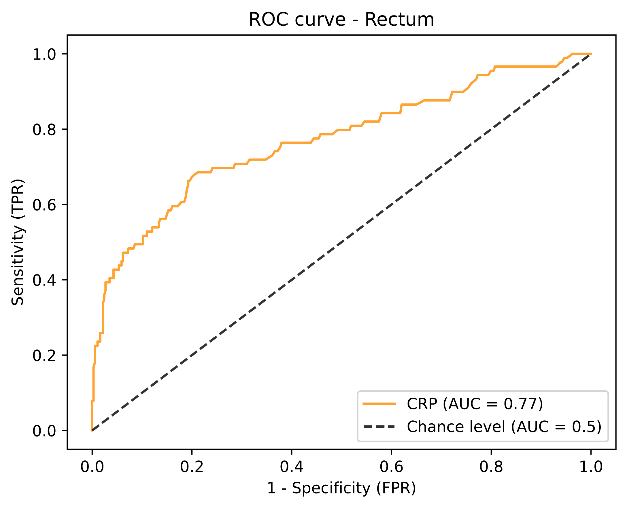

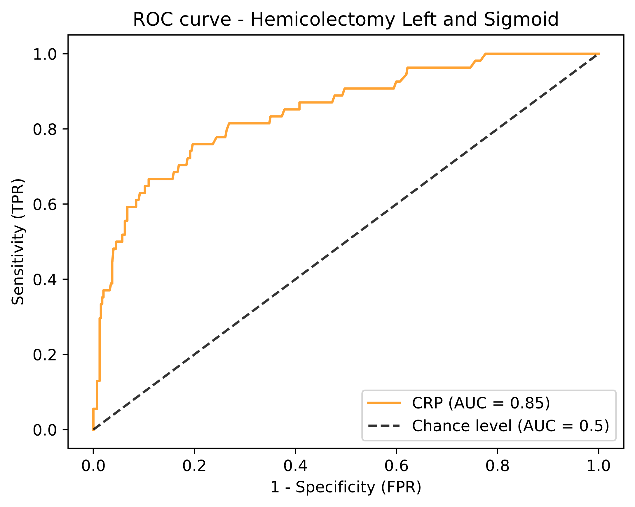


**Figure 7.a-t.** ROC curves with corresponding AUC for all patient and surgery characteristic subgroups

**Table 3.** Summary of optimal cut-off values, specificity and AUS corresponding to a sensitivity of 0.80 for all patient and surgery characteristic subgroups

|  | Optimal cut-off value | Specificity | AUC |
| --- | --- | --- | --- |
| Total population | 114 | 0.58 | 0.78 |
| Age ≤ 65 | 101 | 0.58 | 0.79 |
| Age 66-69 | 109 | 0.49 | 0.74 |
| Age 70-74 | 112 | 0.53 | 0.73 |
| Age ≥75 | 115 | 0.39 | 0.74 |
| Male | 117 | 0.56 | 0.77 |
| Female | 101 | 0.54 | 0.78 |
| Normal/ underweight | 104 | 0.60 | 0.79 |
| Overweight | 115 | 0.58 | 0.80 |
| Obese | 113 | 0.48 | 0.73 |
| ASA I | 66 | 0.41 | 0.76 |
| ASA II | 112 | 0.58 | 0.78 |
| ASA III | 114 | 0.53 | 0.74 |
| ASA IV | 113 | 0.50 | 0.88 |
| Laparoscopic | 112 | 0.64 | 0.79 |
| Robot Assisted | 84 | 0.41 | 0.72 |
| Open | 150 | 0.60 | 0.75 |
| Converted | 109 | 0.39 | 0.66 |
| Hemicolectomy right/ transversum | 122 | 0.54 | 0.74 |
| Hemicolectomy left/ sigmoid | 127 | 0.73 | 0.84 |
| Rectum | 91 | 0.48 | 0.77 |

**Table 4.** Uni- and multivariable logistic regression analysis for predicting major complications with POD 3 CRP optimal cut-off value of 114 mg/L

| Variables | Univariable OR (95% CI) | P- value | Multivariable OR (95% CI) | P-value |
| --- | --- | --- | --- | --- |
| Cutoff Value POD 3 CRP > 114 | 5.58(3.99-7.80) | **<0.001** | 5.29(3.55-7.90) | **<0.001** |
| Age ≤ 65 |  |  | Reference |  |
| Age 66-69 |  |  | 1.73 (1.06-2.83) | **0.030** |
| Age 70-74 |  |  | 1.00 (0.60-1.68) | 0.996 |
| Age ≥75 |  |  | 0.91 (0.57-1.46) | 0.687 |
| Male |  |  | Reference |  |
| Female |  |  | 1.18 (0.82-1.69) | 0.377 |
| Normal/ underweight |  |  | Reference |  |
| Overweight |  |  | 0.68 (0.46-1.01) | 0.053 |
| Obese |  |  | 0.54 (0.34-0.86) | **0.031** |
| Non smoker |  |  | Reference |  |
| Former smoker |  |  | 0.94 (0.64-1.39) | 0.756 |
| Current smoker |  |  | 1.39(0.84-2.31) | 0.198 |
| Non alcohol consumption |  |  | Reference |  |
| Alcohol <1 glass a day |  |  | 0.87 (0.58-1.31) | 0.509 |
| Alcohol ≥1 glass a day |  |  | 1.16 (0.73-1.86) | 0.527 |
| ASA I |  |  | Reference |  |
| ASA II |  |  | 1.90 (0.77 – 4.73) | 0.166 |
| ASA III |  |  | 3.35 (1.31- 8.58) | **0.012** |
| ASA IV |  |  | 19.07 (4.08-89.20) | **<0.001** |
| Laparoscopic |  |  | Reference |  |
| Robot Assisted |  |  | 1.08 (0.50-2.33) | 0.85 |
| Open |  |  | 1.67 (1.11 – 2.51) | **0.013** |
| Converted |  |  | 1.99 (1.26-3.15) | **0.003** |
| Hemicolectomy right/ transversum |  |  | Reference |  |
| Hemicolectomy left/ sigmoid |  |  | 1.00 (0.65-1.54) | 0.999 |
| Rectum |  |  | 1.92 (1.28-.88) | **0.002** |

**Table 5.** Clavien Dindo Classification

| Grade | Definition |
| --- | --- |
| 0 | No complication observed |
| I | Any deviation from the normal postoperative course without the need for pharmacological treatment or surgical, endoscopic, and radiological interventions. Allowed therapeutic regimens are drugs as antiemetics, antipyretics, analgetics, diuretics, electrolytes, and physiotherapy. This grade also includes wound infections opened at the bedside. |
| II | Requiring surgical, endoscopic or radiological intervention |
| IIIa | Intervention not under general anesthesia |
| IIIb | Intervention under general anesthesia |
| IVa | Life-threatening complication requiring intensive care unit management, with single organ dysfunction |
| IVb | Life-threatening complication requiring intensive care unit management, with multiorgan dysfunction |
| V | Death of a patient |
